# Structures and Biological Activities of Secondary Metabolites from *Trichoderma harzianum*

**DOI:** 10.3390/md20110701

**Published:** 2022-11-09

**Authors:** Rui Guo, Gang Li, Zhao Zhang, Xiaoping Peng

**Affiliations:** 1 Department of Natural Medicinal Chemistry and Pharmacognosy, School of Pharmacy, Qingdao University, Qingdao 266071, China; 2 Department of Hand and Foot Surgery, Affiliated Hospital of Qingdao University, Qingdao 266003, China

**Keywords:** natural products, *Trichoderma harzianum*, marine sources, bioactivity, secondary metabolites

## Abstract

The biocontrol fungus *Trichoderma harzianum*, from both marine and terrestrial environments, has attracted considerable attention. *T. harzianum* has a tremendous potential to produce a variety of bioactive secondary metabolites (SMs), which are an important source of new herbicides and antibiotics. This review prioritizes the SMs of *T. harzianum* from 1988 to June 2022, and their relevant biological activities. Marine-derived SMs, especially terpenoids, polyketides, and macrolides compounds, occupy a significant proportion of natural products from *T. harzianum*, deserving more of our attention.

## 1. Introduction

The unique marine environment with high pressure, high salinity, and low temperature, breeds unique marine microorganisms [1,2]. Secondary metabolites obtained from marine-derived fungi have attracted considerable attention in recent years for potential use in the discovery of unique structures and diverse biological properties [3,4]. 

The biocontrol fungi *Trichoderma* spp. (sordariomycetes) are widely spread in the environment [5], such as in the ocean. With the deepening of marine science and technology exploration, more and more *Trichoderma* sp. strains have been discovered from marine sources. From marine and terrestrial environments, there are no fewer than 250 *Trichoderma* species discovered so far [6]. *Trichoderma* species are famous for producing plentiful secondary metabolites [7]. Among them, *Trichoderma harzianum* probably contributed the most secondary metabolites (SMs) originating from *Trichoderma* species [8,9]. The SMs from *T. harzianum* showed antifungal activity [10]. Additionally, cytotoxicity [11] and antimicrobial activity [12], and so on, have also been found in its SMs.

The SMs of *T. harzianum* have not been summarized in detail or systematically. Up to now, nearly 200 compounds of *T. harzianum* have been reported. The secondary metabolites of *T. harzianum* include terpenoids, polyketides, peptides, alkaloids, and lactones. Herein, this review reports the isolated compounds of *T. harzianum* and their bioactivities. Furthermore, details of the source organisms were analyzed for marine and terrestrial sources. A total number of 180 compounds are presented in this review with 58 cited references. These references cover the time period from 1988 to June 2022. 

## 2. Structural and Biological Activity Studies

### 2.1. Terpenoids

**Figure 1:**
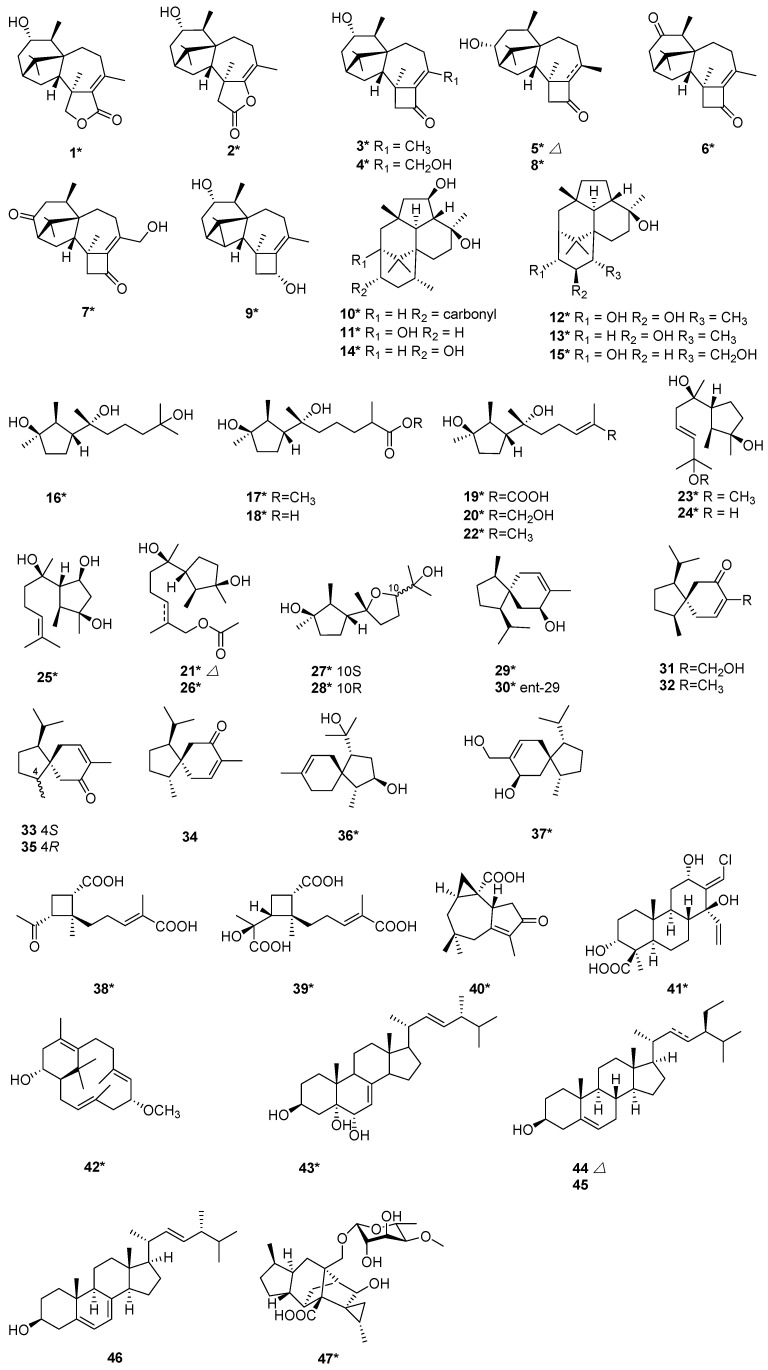
Chemical structures of terpenoids (**1**–**47**) from *T. harzianum.* * Means marine source compounds.

Seven new potent phytotoxic harziane diterpenes harzianelactones A and B (**1** and **2**), harzianones A–D (**3**–**6**) and harziane (**9**) were isolated from the soft coral-derived fungus *T. harzianum* XS-20090075 [13]. Compounds **1** and **2** belonged to a unique class of terpenes with a 6-5-7-5-fused carbocyclic core and a lactone ring. Harzianones A–D (**3**–**6**) consisted of a fused tetracyclic 6-5-7-4-fused tetra-cyclic skeleton. Chemical epigenetic manipulation was applied to activate silent genes of *T. harzianum* XS-20090075 by appending a histone deacetylase (HDAC) inhibitor. With this experimental technique, two new diterpenoids harzianone E (**7**) and harzianolic acid A (**41**), and one new sesquiterpenoid 3,7,11-trihydroxy-cycloneran (**16**) were isolated from the same strain *T. harzianum* XS-20090075. At the same time, 11 known sesquiterpenoids, methyl 3,7-dihydroxy-15-cycloneranate (**17**), catenioblinc (**18**), ascotrichic acid (**19**), cyclonerotriol (**20**), (10*E*)-12-acetoxy-10-cycloneren-3,7-diol (**21**), cyclonerodiol (**22**), cyclonerodiol oxide (**27**), epicyclonerodiol oxide (**28**), ent-trichoacorenol (**29**), trichoacorenol (**30**), and ophioceric acid (**40**) were isolated from *T. harzianum* XS-20090075 [14]. It was the first time for obtaining cleistanthane diterpenoid from *T. harzianum* XS-20090075. Trichodermanins C–H (**10**–**15**) were new diterpenes with a rare fused 6-5-6-6 ring system, and have been isolated from a fungus *T. harzianum* OUPS-111D-4 [15,16]. This strain was separated from a piece of sponge *Halichondria okadai*. Compounds **10**–**15** were evaluated for their cytotoxicity by using murine P388 leukemia, human HL-60 leukemia, and murine L1210 leukemia cell lines. Compound **10** with a fused 6-5-6-6 ring system exhibited potent cytotoxic activity [15], and compounds **12** and **13** exhibited modest activity [16]. Six new terpenes, including one harziane diterpene, 3*R*-hydroxy-9*R*,10*R*-dihydroharzianone (**8**), three cyclonerane sesquiterpenes, methyl 3,7-dihydroxy-15-cycloneranate (**17**), 11-methoxy-9-cycloneren-3,7-diol (**23**), 10-cycloneren-3,5,7-triol (**25**), and one acorane sesquiterpene, 8-acoren-3,11-diol (**36**), and one cyclonerane 11*R*-methoxy-5,9,13-proharzitrien-3-ol (**42**), together with four known sesquiterpenes, cyclonerodio (**22**), 9-cycloneren-3,7,11-triol (**24**), trichoacorenol (**30**) and trichoacorenol B (**37**) were isolated from *T. harzianum* X-5 [17]. The strain X-5 was an endophytic fungus isolated from the marine brown alga *Laminaria japonica*. The above six new compounds (**8, 17, 23, 25, 36,** and **42**) were evaluated to inhibit four marine phytoplankton species and four marine-derived pathogenic bacteria [17]. Compounds **23** and **42** exhibited potent inhibition activity [17]. Harzianoic acid A (**38**) is a sesquiterpene, and harzianoic acid B (**39**) is a norsesquiterpene with a cyclobutane nucleus. They were isolated from a sponge-isolated fungus, *T. harzianum* LZDX-32-08 [18], and were found to have new natural scaffolds to exert anti-HCV activity for their capability to inhibit multi-targets, including those for virus replication and entry [18]. (10*E*)-12-Acetoxy-10-cycloneren-3,7-diol (**21**) and 12-acetoxycycloneran-3,7-diol (**26**) were two new cyclonerane sesquiterpenoids, which were isolated from the marine sediment-derived fungus *T. harzianum* P1-4 [9]. A new acorane-type sesquiterpene, 15-hydroxyacorenone (**31**), was isolated from *T. harzianum* [19], together with acorenone (**32**), acorenone-B (**33**), 4-epiacorenone (**34**), and 4-epiacorenone-B (**35**). Stigmasta-7,22-dien-3*β*,5*α*,6*α*-triol (**43**) was isolated from *T. harzianum* XS-20090075, cultivated by the Czapekʹs culture [20]. Compound **43** exhibited antifouling activity with an EC_50_ value of 39.2 μg/mL and Topo I inhibitory activity with an MIC value of 50.0 μM [20]. Two fungal strains of *T. harzianum* T-4 and *T. harzianum* T-5 were obtained from Palampur, Himachal Pradesh (India). Stigmasterol (**44**) and *β*-sitosterol (**45**) were isolated from *T. harzianum* T-4 [21]. Ergosterol (**46**) was isolated from *T. harzianum* T-5 [21]. Trichosordarin A (**47**), a unique norditerpene aglycone, was isolated from *T. harzianum* R5 [22]. Compound **47** was toxic to the marine zooplankton *Artemia salina* with an LC_50_ value of 233 µM [22] (Figure 1).

### 2.2. Polyketides

The fermentation of a sponge-associated fungus *T. harzianum* HMS-15-3 led to the isolation of four pairs of new C_13_ lipid enantiomers harzianumols A–H (**48**–**55**) [23]. Four polyketides, trichoharzin B (**56**), methyl-trichoharzin (**57**), trichoharzin (**58**), and eujavanicol A (**59**), were isolated from *T. harzianum* XS-20090075 [20], which was fermented in rice medium by one strain many compounds (OSMAC) strategy. New naphthalene compound **57**, and known naphthalene compound **58** exhibited antifouling activity with the EC_50_ values of 29.8 and 35.6 μg/mL [20]. Six new tandyukisins, tandyukisins A–F (**60**–**65**), were isolated from *T. harzianum* OUPS-111D-4 [11,24,25], which were initially derived from the sponge *Halichondria okadai*. Among the tandyukisins A–F (**60**–**65**), compounds **60**, **64** and **65** exhibited cytotoxicity against murine P388 leukemia, human HL-60 leukemia, and murine L1210 leukemia cell lines inferior to the control 5-fluorouracil [24]. Compounds **61**–**63** showed slightly selective growth inhibition against the central nervous system cancer SNB-75 cell line in the HCC panel [25]. Compounds **64** and **65** exhibited significant cytotoxicity against the cancer cell lines P388, HL-60, and L1210 [24]. The structure-activity relationship may be relevant to the terminals of the side chains. *T. harzianum* T-4 was obtained from Palampur, Himachal Pradesh in India, and a polyketide palmitic acid (**66**) was isolated from the T-4 [21]. Harzianum A (**67**), was a new trichothecene isolated from the soil-borne fungus *T. harzianum* in 1994 [26]. Harziphilone (**68**) was a new polyketide isolated from *T. harzianum* WC 47695 [27], which was isolated from sandy soil with plant debris collected in Fort Lauderdale. The REV/RRE binding assay and HIV assay revealed that compound **68** showed inhibitory activity against REV-protein binding to RRE RNA with IC_50_ values of 2.0 μM. In contrast, this compound did not show protection against HIV infection at concentration levels up to 200 μg/mL. The cytotoxicity assay on the murine tumor cell line M-109 showed that **68** exhibited cytotoxicity at 38 μM [27]. Seven polyketides, keto triol 3 (**69**), keto diol 7 (**70**), keto diol 6 (**71**), keto diol 8 (**72**), triacetate 9 (**73**), triol 10 (**74**) and acetal diol 2 (**75**) were isolated from *T. harzianum* [28]. One new trichoharzin (**58**), and two known compounds, tribenzoate (**76**) and triacetate (**77**), were isolated from *T. harzianum* Rifai in 1993 [29]. A new polyketide, T22azaphilone (**78**), was isolated from *T. harzianum* T22 [30]. A new compound, trichoharzianol (**79**), isolated from *T. harzianum* F031, exhibited antifungal activity against *Colletotrichum gloeosporioides* with a MIC of 128 μg/mL [31]. Three novel polyketides trichodenones A–C (**80**–**82**) were isolated from *T. harzianum* OUPS-N115 [32]. This strain was separated from the sponge *Halichondria okadai*. Trichodenones A–C (**80**–**82**) showed cytotoxicities against P388 cell line with the ED_50_ values of 0.21, 1.21, and 1.45 μg/mL, respectively. Homodimericin A (**83**) was isolated from *T. harzianum* WC13 [33,34]. In their model, compound **83** was the biologically inert aftermath of a fungal counter to a bacterial attack. The discovery of cryptenol (**84**) from *T. harzianum* WC13 [34] indicated that the interactions among microbes in a termite nest were not bipartite but a multipartite system.

**Figure 2:**
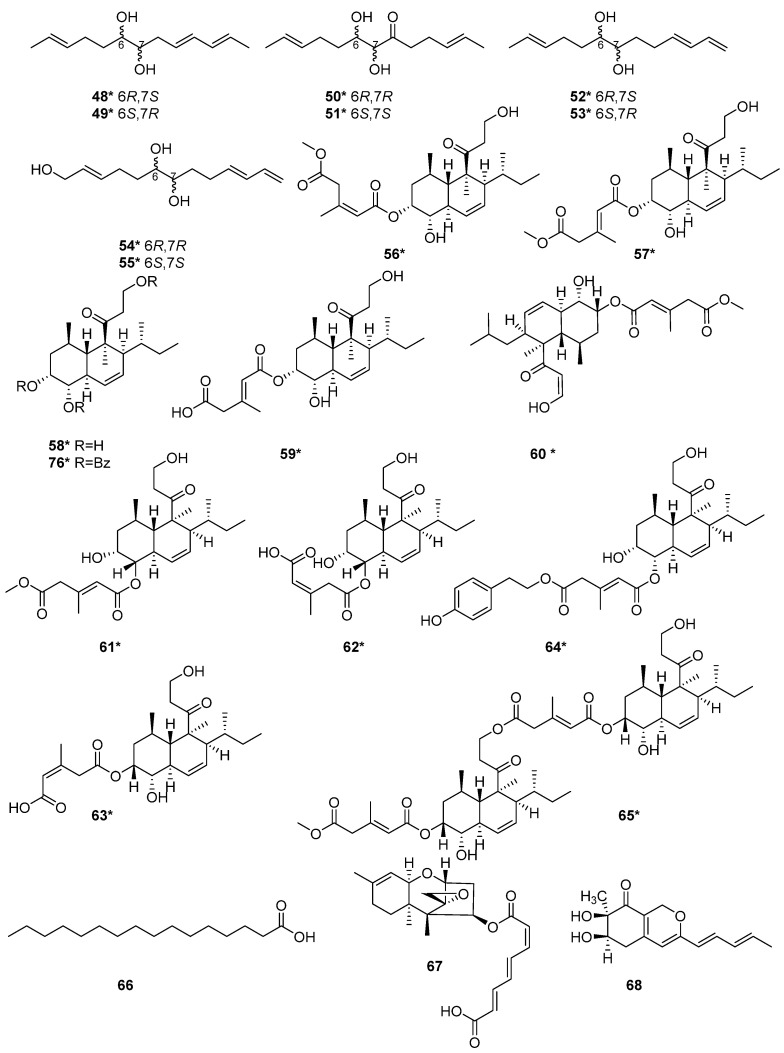
Chemical structures of polyketides (**48**–**68** and **76**) from *T. harzianum.* * Means marine source compounds.

**Figure 3:**
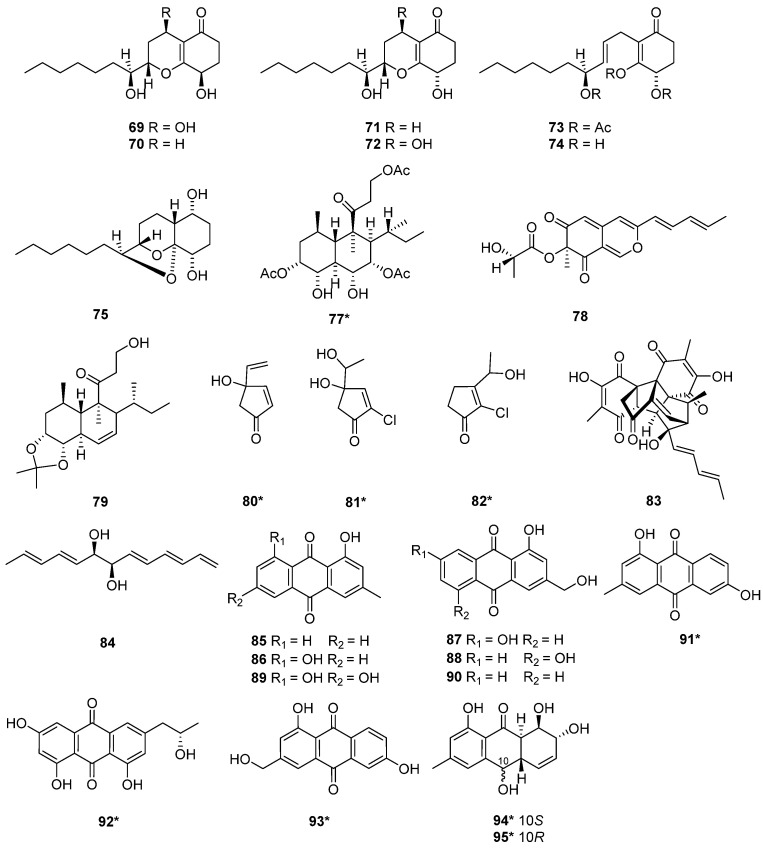
Chemical structures of polyketides (**69**–**75** and **77**–**95**) from *T. harzianum.* * Means marine source compounds.

The structure and activity relationships of anthraquinones (AQs) in *T. harzianum* have been studied. AQs represent an important class of SMs occurring in *T. harzianum* strains, which exhibited a variety of biological functions [12]. The alkylating functionalities in the AQs maximize the anticancer activity by binding tightly with DNA to disrupt the DNA function [35]. Moreover, anthraquinone derivatives were proposed to have an anticancer function by inhibiting protein kinase CK2 [36]. Pachybasin (**85**) and chrysophanol (**86**) were isolated from *T. harzianum* ETS 323 [37]. 1,7-Dihydroxy-3-hydroxymethyl-9,10-anthraquinone (**87**), 1,5-dihydroxy-3-hydroxymethyl-9,10-anthraquinone (**88**), emodin (**89**), and *ω*-hydroxypachybasin (**90**) were isolated from *T. harzianum* strain Th-R16 [38]. These compounds exhibited effective antifungal activity against *Botrytis cinerea* (Ascomycete) and *Rhizoctonia solani* (Basidiomycete). At a 500 μg/mL concentration, compound **88** showed comparatively higher activity against *R. solani* and *B. cinerea* than **89** [38]. Phomarin (**91**), (+)-2′*S*-isorhodoptilometrin (**92**), 1,6-dihydroxy-3-(hydroxymethyl)anthracene-9,10-dione (**93**), harzianumnone A (**94**) and harzianumnone B (**95**) were isolated from the soft coral-derived fungus *T. harzianum* XS-20090075 [12]. Compounds **94** and **95** were identified as a pair of epimers, the first example of hydroanthraquinones from *T. harzianum* XS-20090075. Compound **92** with Topo I inhibition activity, was further assessed for cytotoxic activity against human tumor cell lines. It exhibited cytotoxic activity against HepG2 cell line with an IC_50_ value of 2.10 µM, and showed cytotoxicity against Hela cell with an IC_50_ value of 8.59 µM [12] (Figure 2 and Figure 3).

### 2.3. Peptides

**Figure 4:**
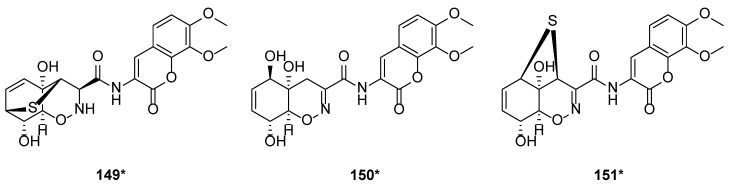
Chemical structures of peptides (**149**–**151**) from *T. harzianum.* * Means marine source compounds.

Peptaibols are linear antibiotic peptides consisting of 5 to 20 amino acids [39]. It could be biosynthesized by *T. harzianum*. Peptaibols were characterized by the structures of alpha-aminoisobutyric acid (Aib), and C-terminal hydroxylated amino acid. Two new series peptaibols, trichokindins (TKs) and trichorozins (TZs), were isolated from *T. harzianum* collected at Nara in Japan. TKs and TZs comprised 18 and 11 amino acid residues, respectively, while TKs were rich in isovaline (Iva). TK-VII (**106**) is the most hydrophobic of TKs with 18-residue peptides. Compound **106** induced Ca^2+^-dependent catecholamine secretion from bovine adrenal medullary chromaffin cells [40]. TKs (**96**–**106**), with a single peak on HPLC and typical IR absorptions at 3300, 1600, and 1530 cm^−1^, were confirmed as peptaibols by polarization transfer spectra [40]. With incubating 10 μM of TK-VII (**106**), 27% of the total catecholamines in bovine adrenal chromaffin cells were secreted in the presence of the Ca^2+^. In contrast, only 5% of the total catecholamines were secreted without Ca^2+^ [40]. Hydrophobicity is vital to the interaction between membranes and peptaibols [41]. HB I (**107**) was isolated from *T. harzianum* M-903603 [42]. Trichorzins HA (**108**–**113**) and MA (**114**–**116**) were isolated from *T. harzianum* M-903602 and *T. harzianum* M-922835, respectively. Compounds **108**–**116** are a series of 18-residue peptides [43]. Bioassays on the antifungal activity of trichorzins and harzianins on the phytopathogenic fungus *Sclerotium cepivorum* revealed that trichorzins were more potent (75% inhibition at 100 μg/mL) than harzianins (40% inhibition at 100 μg/mL) [44]. Research on the structured-activity relationships (SARs) revealed that the peptide chain length and superhydrophobicity played an essential part in the peptide/membrane interaction and the subsequent permeability by perturbing the ironic balance of the cell [44]. As new membrane-modifying peptides isolated from *T. harzianum*, trichorozins I–IV (**117**–**120**), belonged to peptaibols with 11 residues. It was reported that compounds **117**–**120** exhibited voltage-dependent ion channel-like activity in lipid bilayers [45]. Eleven peptides were isolated from *T. harzianum* M-903603, and named harzianins HC (**121**–**131**) [46]. The detailed study of such proline-rich 14-residue peptaibols revealed that harzianins HC increased the permeability of liposomes and improved voltage-dependent conductance [46]. An exogenous amino acid supply simplified the microheterogeneous peptide mixtures when Aib, Glu, or Arg was added to the fermentation media of *T. harzianum* M-902608. Harzianin PC_U_4 (**132**), trichorzin PA_U_4 (**133**), trichorzin PA II (**134**), trichorzin PA IV–VIII (**135**–**139**) and trichorzin PA IX (**140**) were isolated from this *T. harzianum* M-902608 [47]. When cultured in the Aib-enriched media, compounds **132** and **133** were isolated, while trichorzins PA was obtained from the standard culture media [47]. Trichorzianines A (TA) and B (TB) are peptaibols isolated from *T. harzianum*. TA IIIc (**141**) induced the growth inhibition and lysis of the amoeba *Dictyostelium* [48]. With the aid of positive ion FAB mass spectrometry, COSY and NOESY experiments, seven peptides of trichorzianines B isolated from *T. harzianum* were identified, and these peptides included trichorzianine TB IIa (**142**), trichorzianine TB IIIc (**143**), trichorzianine TB IVb (**144**), trichorzianine TB Vb (**145**), trichorzianine TB VIa (**146**), trichorzianine TB VIb (**147**) and trichorzianine TB VII (**148**) [49]. From a mangrove-derived fungus, *T. harzianum* D13, a novel heterocyclic dipeptide trichodermamide G (**149**), two known biogenetically related compounds, trichodermamide A (**150**) and aspergillazin A (**151**) were isolated. A unique sulfur bridge was observed in the structures of compounds **149** and **151** [50] (Table 1 and Figure 4).

### 2.4. Alkaloids

**Figure 5:**
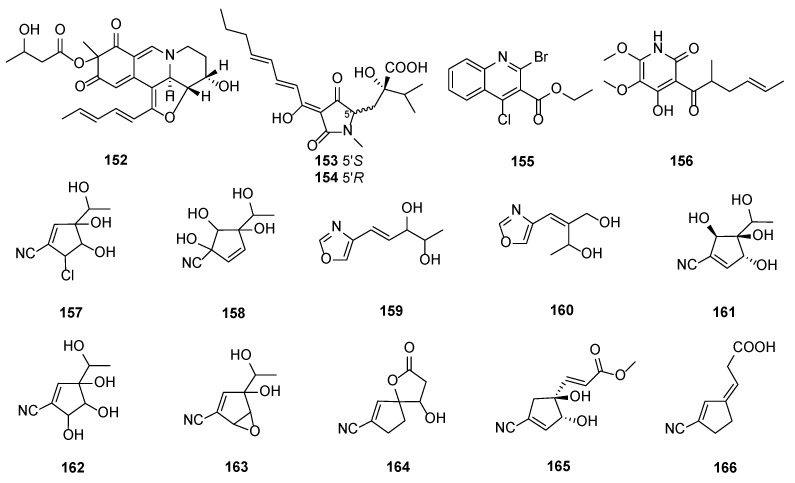
Chemical structures of alkaloids (**152**–**166**) from *T. harzianum.*

Fleephilone (**152**), a new HIV REV/RRE binding inhibitor, was produced by *T. harzianum* WC 47695 [27] isolated from sandy soil with plant debris collected in Fort Lauderdale, FL, USA. Compound **152** showed inhibitory activity against REV-protein binding to RRE RNA with an IC_50_ value of 7.6 μM, and exhibited no protection against HIV infection at concentrations up to 200 μg/mL. Harzianic acid (**153**) was isolated from *T. harzianum* SY-307, which exhibited antimicrobial activity against *Pasteurella piscicida* sp. 6395 [51]. Isoharzianic acid (**154**), a new stereoisomer of compound **153**, was isolated from the *T. harzianum* strain M10, together with Harzianic acid (HA) [52]. HA was able to promote plant growth and strongly bind iron [52]. An OSMAC approach using multiple culture conditions or co-cultures has been applied to access the chemical diversity of *T. harzianum* XS-20090075 [20]. A new halogenate quinoline natural product, ethyl 2-bromo-4-chloroquinoline-3-carboxylate (**155**), was isolated from *T. harzianum* XS-20090075 [20]. Harzianopyridone (**156**) was isolated from the *T. harzianum* T-5. This strain was obtained from Palampur, Himachal Pradesh, India [21]. Compound **156** inhibited more than 90% growth of *Rhizoctonia solani*, *Sclerotium rolfsii*, and *Fusarium oxysporum* (EC_50_ 35.9–50.2 μg/mL), but was less active than Bavistin [21]. A new oxazole metabolite, MR93A (**159**) was isolated from *T. harzianum* KCTC 0114BP [53], while eight metabolites MR566A (**157**), MR566B (**158**), MR93B (**160**), MR304A (**161**), 1-(1,4,5-trihydroxy-3-isocyanocyclopenten-2-enyl)-ethanol (**162**), 2-hydroxy-4-isocyano-*α* -methyl-6-oxabicyclo[3.1.0]-hex-3-ene-2-methanol (**163**), 4-hydroxy-8-isocyano-1- oxaspiro[4.4]cyclonon-8-en-2-one (**164**), methyl-3-(1,5-dihydroxy-3-isocyanocyclopent- 3-enyl)prop-2-enoate (**165**) and 3-(3′-isocyanocyclopent-2′-eny1idene)propionic acid (**166**) were isolated from *T. harzianum* [54]. MR566A (**157**) strongly inhibited mushroom tyrosinase with an IC_50_ value of 1.72 µM compared with kojic acid with an IC_50_ value of 3.08 µM [55]. Compound **166** exhibited inhibitory activity against mushroom tyrosinase with an IC_50_ value of 0.0014 µM, which was more active than the kojic acid [55] (Figure 5).

### 2.5. Lactones

**Figure 6:**
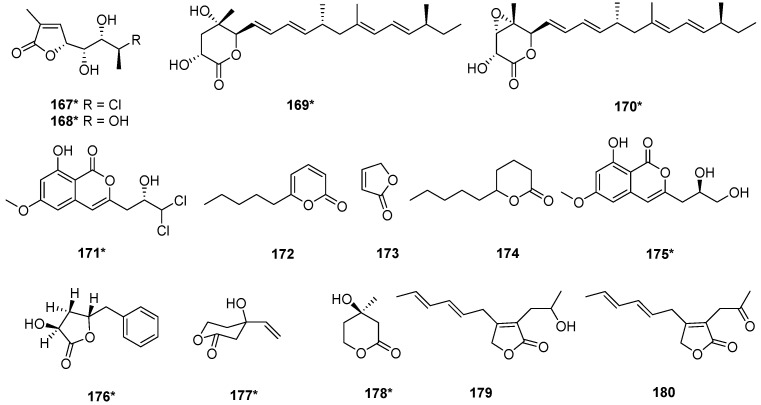
Chemical structures of lactones (**167**–**180**) from *T. harzianum.* * Means marine source compounds.

Two lactones, nafuredins C (**169**) and A (**170**), were isolated from the mangrove-derived fungus *T. harzianum* D13, and the new compound **169** exhibited antifungal activity against *Magnaporthe oryzae*, with an MIC value of 8.63 µM [50]. From *T. harzianum* XS-20090075, four known compounds, xylogibloactones A and B (**167**, and **168**), nafuredin A (**170**), and dichlorodiaportin (**171**) [20,56,57] were isolated. Compound **170** exhibited antifouling activity with the EC_50_ value of 21.4 μg/mL [20]. 6-Pentyl-2*H*-pyran-2-one (**172**) and 2(5*H*)-furanone (**173**) were isolated from *T. harzianum* T-4 [21], while *δ*-decanolactone (**174**) was isolated from *T. harzianum* T-5 [21]. Compound **172**, a volatile organic compound from *T. harzianum* [58], had the ability to inhibit primary root growth and induce lateral root formation. Peniisocoumarin H (**175**) was isolated from the mangrove-derived fungus *T. harzianum* D13 [50]. Two new lactones, harzialactones A (**176**) and B (**177**), together with a known compound *R*-mevalonolactone (**178**), were isolated from *T. harzianum* OUPS-N115 [32]. *T. harzianum* OUPS-N115 was separated from the sponge *Halichondria okadai,* and the cytotoxicity of compounds **176**–**178** against the P388 cell line was tested. The results showed no significant cytotoxicity [32]. Two lactones harzianolide (**179**) and T39butenolide (**180**) were isolated from *T. harzianum* T39 [30] (Figure 6).

**Figure 7:**
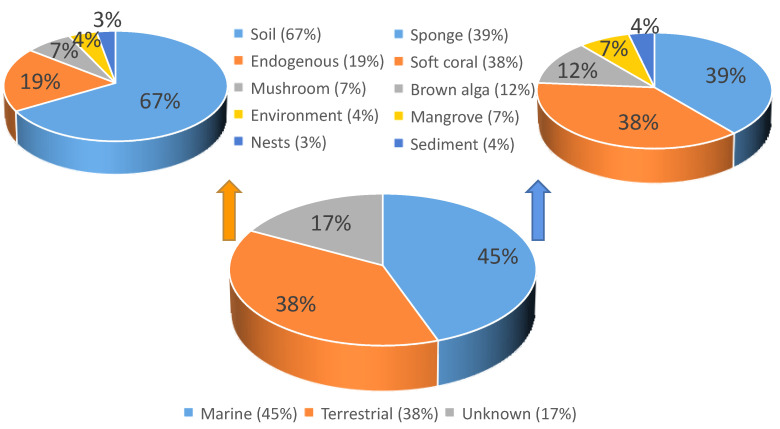
The SMs of *T. harzianum* from marine and terrestrial sources, and its distribution.

**Figure 8:**
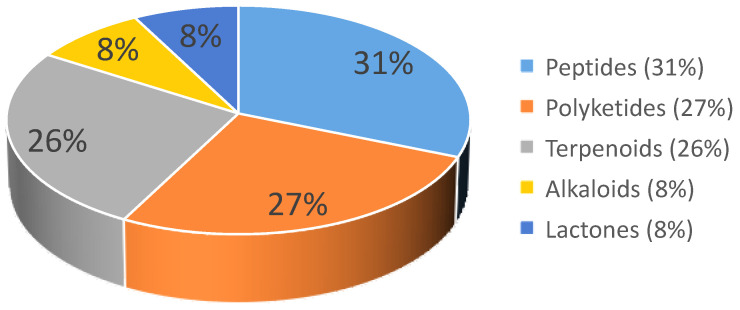
Proportion of SMs obtained from *T. harzianum*.

**Figure 9:**
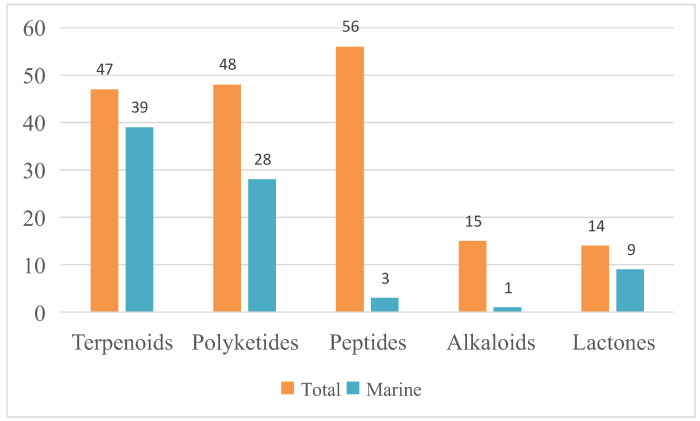
Total numbers and marine source numbers of SMs with each chemical structure type.

**Figure 10:**
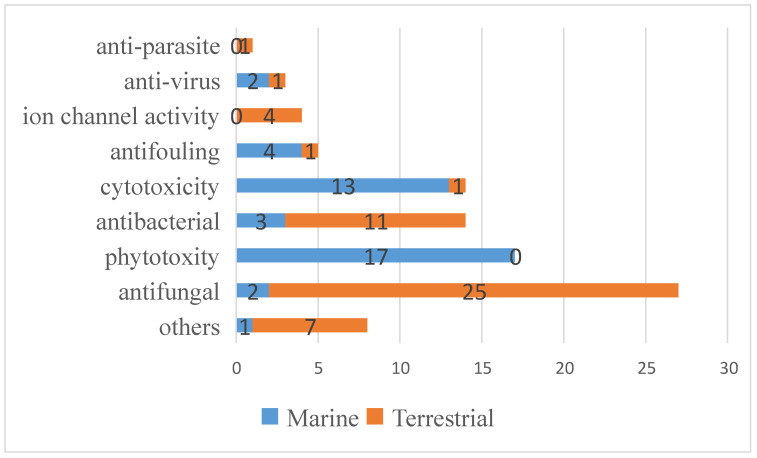
The bioactivities of SMs from *T. harzianum* and the territorial distribution.

All compounds from *T. harzianum* with their biological activities and habitats were summaried in Table 2. As an analysis, the percentage of marine sources and terrestrial sources from the SMs distribution were exhibited, including the specific source ratio (Figure 7). The structure type proportion and the bioactivity distribution of the SMs isolated from *T. harzianum* were also shown (Figure 8, Figure 9 and Figure 10).

## 3. Conclusions

This review covers papers on metabolites isolated from *T. harzianum.* From the SMs’ distribution point of view, marine sources account for 45%, while terrestrial sources were 38%. From marine sources, 31 compounds were from sponges-derived *T. harzianum* strains, 30 compounds were isolated from soft corals-derived *T. harzianum* strains, 10 compounds were from brown alga-derived *T. harzianum* strains, 6 compounds were from mangrove samples-derived *T. harzianum* strains, and 3 compounds were from marine sediment samples. *T. harzianum* strains and their secondary metabolites were mainly derived from sponges (39%) and soft corals (38%). From the terrestrial sources, 46 compounds were purified from soil samples-derived *T. harzianum* strains, 13 compounds were from endogenous and 5 compounds were purified from mushroom-derived fungal strains. Compounds derived from terrestrial soil samples account for 67%. For the structure type proportion of the SMs isolated from *T. harzianum*, the peptides, polyketides, and terpenoids account for 31%, 27%, and 26%, respectively, followed by alkaloids (8%) and lactones (8%). Marine-derived terpenoids and polyketides have 39 and 28 natural products among the 47 and 48 total compounds, respectively. Notably, 91 of the 180 SMs exhibited bioactivities. Antifungal activity was exhibited by 27 natural products, and 17 compounds possessed phytotoxicity activity, while antibacterial and cytotoxicity activity SMs number were all 14. In the research on phytotoxicity and cytotoxic active products, almost all the active natural products were from marine-derived *T. harzianum* strains. Moreover, 120 of the 180 compounds were new.

In summary, organic compounds are abundant in the SMs of *T. harzianum*, they may be used as a fungicide, antibacterial, antineoplastic, and weedicide, both in clinical and agricultural applications. The marine sources molecules (marked * in this paper) with their unique molecular and diverse activities, could be the basis for the development of new drug-forming lead compounds.

## Figures and Tables

**Table 1:** The sequences of peptides (**96**–**148**) from *T. harzianum*.

	Compounds	Sequences of Peptides
**96**	Trichokindin Ia	Ac Aib Ser Ala Aib Aib Gln Iva Leu Aib Ala Aib Aib Pro Leu Aib Aib Gln Ile OH
**97**	Trichokindin Ib	Ac Aib Ser Ala Aib Iva Gln Aib Leu Aib Ala Aib Aib Pro Leu Aib Aib Gln Ile OH
**98**	Trichokindin IIa	Ac Aib Ser Ala Aib Aib Gln Aib Leu Aib Ala Iva Aib Pro Leu Aib Aib Gln Ile OH
**99**	Trichokindin IIb	Ac Aib Ser Ala Aib Iva Gln Iva Leu Aib Ala Aib Aib Pro Leu Aib Aib Gln Leu OH
**100**	Trichokindin IIIa	Ac Aib Ser Ala Aib Aib Gln Iva Leu Aib Ala Iva Aib Pro Leu Aib Aib Gln Leu OH
**101**	Trichokindin IIIb	Ac Aib Ser Ala Aib Iva Gln Aib Leu Aib Ala Iva Aib Pro Leu Aib Aib Gln Leu OH
**102**	Trichokindin IV	Ac Aib Ser Ala Aib Iva Gln Iva Leu Aib Ala Aib Aib Pro Leu Aib Aib Gln Ile OH
**103**	Trichokindin Va	Ac Aib Ser Ala Aib Aib Gln Iva Leu Aib Ala Iva Aib Pro Leu Aib Aib Gln Ile OH
**104**	Trichokindin Vb	Ac Aib Ser Ala Aib Iva Gln Aib Leu Aib Ala Iva Aib Pro Leu Aib Aib Gln Ile OH
**105**	Trichokindin VI	Ac Aib Ser Ala Aib Iva Gln Iva Leu Aib Ala Iva Aib Pro Leu Aib Aib Gln Leu OH
**106**	Trichokindin VII	Ac Aib Ser Ala Aib Iva Gln Iva Leu Aib Ala Iva Aib Pro Leu Aib Aib Gln Ile OH
**107**	Harzianin HB I	Ac Aib Asn Leu Ile Aib Pro Iva Leu Aib Pro Leu OH
**108**	Trichorzin HA I	Ac Aib Gly Ala Aib Aib Gln Aib Val Aib Gly Leu Aib Pro Leu Aib Aib Gln Leu OH
**109**	Trichorzin HA II	Ac Aib Gly Ala Aib Aib Gln Aib Val Aib Gly Leu Aib Pro Leu Aib Iva Gln Leu OH
**110**	Trichorzin HA III	Ac Aib Gly Ala Aib Iva Gln Aib Val Aib Gly Leu Aib Pro Leu Aib Aib Gln Leu OH
**111**	Trichorzin HA V	Ac Aib Gly Ala Aib Iva Gln Aib Val Aib Gly Leu Aib Pro Leu Aib Iva Gln Leu OH
**112**	Trichorzin HA VI	Ac Aib Gly Ala Aib Iva Gln Iva Val Aib Gly Leu Aib Pro Leu Aib Iva Gln Leu OH
**113**	Trichorzin HA VII	Ac Aib Gly Ala Aib Iva Gln Val Val Aib Gly Leu Aib Pro Leu Aib Iva Gln Leu OH
**114**	Trichorzin MA I	Ac Aib Ser Ala Aib Aib Gln Aib Leu Aib Gly Leu Aib Pro Leu Aib Aib Gln Val OH
**1** **15**	Trichorzin MA II	Ac Aib Ser Ala Aib Iva Gln Aib Leu Aib Gly Leu Aib Pro Leu Aib Aib Gln Val OH
**1** **16**	Trichorzin MA III	Ac Aib Ser Ala Aib Iva Gln Iva Leu Aib Gly Leu Aib Pro Leu Aib Aib Gln Val OH
**1** **17**	Trichorozin I	Ac Aib Asn Ile Leu Aib Pro Ile Leu Aib Pro Val OH
**1** **18**	Trichorozin II	Ac Aib Gln Ile Leu Aib Pro Ile Leu Aib Pro Val OH
**1** **19**	Trichorozin III	Ac Aib Asn Ile Leu Aib Pro Ile Leu Aib Pro Leu OH
**1** **20**	Trichorozin IV	Ac Aib Gln Ile Leu Aib Pro Ile Leu Aib Pro Leu OH
**1** **21**	Harzianin HC I	Ac Aib Asn Leu Aib Pro Ser Val Aib Pro Aib Leu Aib Pro Leu OH
**1** **22**	Harzianin HC III	Ac Aib Asn Leu Aib Pro Ser Val Aib Pro Iva Leu Aib Pro Leu OH
**1** **23**	Harzianin HC VI	Ac Aib Asn Leu Aib Pro Ala Val Aib Pro Aib Leu Aib Pro Leu OH
**1** **24**	Harzianin HC VIII	Ac Aib Asn Leu Aib Pro Ala Val Aib Pro Iva Leu Aib Pro Leu OH
**1** **25**	Harzianin HC IX	Ac Aib Asn Leu Aib Pro Ala Ile Aib Pro Iva Leu Aib Pro Leu OH
**1** **26**	Harzianin HC X	Ac Aib Gln Leu Aib Pro Ala Val Aib Pro Iva Leu Aib Pro Leu OH
**1** **27**	Harzianin HC XI	Ac Aib Asn Leu Aib Pro Ser Ile Aib Pro Aib Leu Aib Pro Leu OH
**1** **28**	Harzianin HC XII	Ac Aib Asn Leu Aib Pro Ser Ile Aib Pro Iva Leu Aib Pro Leu OH
**1** **29**	Harzianin HC XIII	Ac Aib Gln Leu Aib Pro Ser Ile Aib Pro Iva Leu Aib Pro Leu OH
**1** **30**	Harzianin HC XIV	Ac Aib Asn Leu Aib Pro Ala Ile Aib Pro Aib Leu Aib Pro Leu OH
**1** **31**	Harzianin HC XV	Ac Aib Gln Leu Aib Pro Ala Ile Aib Pro Iva Leu Aib Pro Leu OH
**1** **32**	Harzianin PC_U_4	Ac Aib Asn Leu Aib Pro Ser Ile Aib Pro Aib Leu Aib Pro Val OH
**1** **33**	Trichorzin PA_U_4	Ac Aib Ser Ala Aib Aib Gln Aib Val Aib Gly Leu Aib Pro Leu Aib Aib Gln Trp OH
**1** **34**	Trichorzin PA II	Ac Aib Ser Ala Aib Iva Gln Aib Val Aib Gly Leu Aib Pro Leu Aib Aib Gln Trp OH
**1** **35**	Trichorzin PA IV	Ac Aib Ser Ala Aib Iva Gln Iva Val Aib Gly Leu Aib Pro Leu Aib Aib Gln Trp OH
**1** **36**	Trichorzin PA V	Ac Aib Ser Ala Iva Iva Gln Aib Val Aib Gly Leu Aib Pro Leu Aib Aib Gln Trp OH
**1** **37**	Trichorzin PA VI	Ac Aib Ser Ala Aib Iva Gln Aib Val Aib Gly Leu Aib Pro Leu Aib Aib Gln Phe OH
**1** **38**	Trichorzin PA VII	Ac Aib Ser Ala Iva Iva Gln Aib Val Aib Gly Leu Aib Pro Leu Aib Aib Gln Trp OH
**1** **39**	Trichorzin PA VIII	Ac Aib Ser Ala Aib Iva Gln Iva Val Aib Gly Leu Aib Pro Leu Aib Aib Gln Phe OH
**1** **40**	Trichorzin PA IX	Ac Aib Ser Ala Iva Iva Gln Aib Val Aib Gly Leu Aib Pro Leu Aib Aib Gln Phe OH
**1** **41**	Trichorzianine TA IIIc	Ac Aib Ala Ala Aib Aib Gln Aib Aib Aib Ser Leu Aib Pro Val Aib Ile Gln Gln Trp OH
**1** **42**	Trichorzianine TB IIa	Ac Aib Ala Ala Aib Aib Gln Aib Aib Aib Ser Leu Aib Pro Leu Aib Ile Gln Glu Trp OH
**1** **43**	Trichorzianine TB IIIc	Ac Aib Ala Ala Aib Aib Gln Aib Aib Aib Ser Leu Aib Pro Val Aib Ile Gln Glu Trp OH
**1** **44**	Trichorzianine TB IVb	Ac Aib Ala Ala Aib Iva Gln Aib Aib Aib Ser Leu Aib Pro Val Aib Ile Gln Glu Trp OH
**1** **45**	Trichorzianine TB Vb	Ac Aib Ala Ala Aib Aib Gln Aib Aib Aib Ser Leu Aib Pro Leu Aib Ile Gln Glu Phe OH
**1** **46**	Trichorzianine TB VIa	Ac Aib Ala Ala Aib Iva Gln Aib Aib Aib Ser Leu Aib Pro Leu Aib Ile Gln Glu Phe OH
**1** **47**	Trichorzianine TB VIb	Ac Aib Ala Ala Aib Aib Gln Aib Aib Aib Ser Leu Aib Pro Val Aib Ile Gln Glu Phe OH
**1** **48**	Trichorzianine TB VII	Ac Aib Ala Ala Aib Iva Gln Aib Aib Aib Ser Leu Aib Pro Val Aib Ile Gln Glu Phe OH

**Table 2:** The bioactivities and habitats of SMs (**1**–**180**) from *T. harzianum*.

Compounds	Bioactivities	Habitats	Refs
Harzianelactone A (**1**) *	Phytotoxicity	Soft coral	[13]
Harzianelactone B (**2**) *	phytotoxicity	Soft coral	[13]
Harzianone A (**3**) *	phytotoxicity	Soft coral	[13]
Harzianone B (**4**) *	phytotoxicity	Soft coral	[13]
Harzianone C (**5**) *	phytotoxicity	Soft coral	[13]
Harzianone D (**6**) *	phytotoxicity	Soft coral	[13]
Harzianone E (**7**) *	Antibacterial	Soft coral	[14]
3*R*-Hydroxy-9*R*,10*R*-dihydroharzianone (**8**) *	phytotoxicity	Brown alga	[17]
Harziane (**9**) *	phytotoxicity	Soft coral	[13]
Trichodermanin C (**10**) *	Cytotoxicity	Sponge	[15,16]
Trichodermanin D (**11**) *	—	Sponge	[15,16]
Trichodermanin E (**12**) *	Cytotoxicity	Sponge	[15,16]
Trichodermanin F (**13**) *	Cytotoxicity	Sponge	[15,16]
Trichodermanin G (**14**) *	—	Sponge	[15,16]
Trichodermanin H (**15**) *	—	Sponge	[15,16]
3,7,11-Trihydroxy-cycloneran (**16**) *	—	Soft coral	[14]
Methyl 3,7-dihydroxy-15-cycloneranate (**17**) *	Antibacterial	Soft coral	[14]
	phytotoxicity	Brown alga	[17]
Catenioblinc (**18**) *	—	Soft coral	[14]
Ascotrichic acid (**19**) *	—	Soft coral	[14]
Cyclonerotriol (**20**) *	—	Soft coral	[14]
(10*E*)-12-Acetoxy-10-cycloneren-3,7-diol (**21**) *	—	Sediment	[9]
	—	Soft coral	[14]
Cyclonerodiol (**22**) *	—	Soft coral	[14]
	phytotoxicity	Brown alga	[17]
11-Methoxy-9-cycloneren-3,7-diol (**23**) *	phytotoxicity	Brown alga	[17]
9-Cycloneren-3,7,11-triol (**24**) *	phytotoxicity	Brown alga	[17]
10-Cycloneren-3,5,7-triol (**25**) *	phytotoxicity	Brown alga	[17]
12-Acetoxycycloneran-3,7-diol (**26**) *	—	Sediment	[9]
Cyclonerodiol oxide (**27**) *	—	Soft coral	[14]
Epicyclonerodiol oxide (**28**) *	—	Soft coral	[14]
ent-Trichoacorenol (**29**) *	—	Soft coral	[14]
Trichoacorenol (**30**) *	—	Soft coral	[14]
	phytotoxicity	Brown alga	[17]
15-Hydroxyacorenone (**31**)	—	Mushroom	[19]
Acorenone (**32**)	—	Mushroom	[19]
Acorenone-B (**33**)	—	Mushroom	[19]
4-Epiacorenone (**34**)	—	Mushroom	[19]
4-Epiacorenone-B (**35**)	—	Mushroom	[19]
8-Acoren-3,11-diol (**36**) *	phytotoxicity	Brown alga	[17]
Trichoacorenol B (**37**) *	phytotoxicity	Brown alga	[17]
Harzianoic acid A (**38**) *	Antivirus	Sponge	[18]
Harzianoic acid B (**39**) *	Antivirus	Sponge	[18]
Ophioceric acid (**40**) *	—	Soft coral	[14]
Harzianolic acid A (**41**) *	—	Soft coral	[14]
11*R*-Methoxy-5,9,13- proharzitrien-3-ol (**42**) *	phytotoxicity	Brown alga	[17]
Stigmasta-7,22-dien-3*β*,5*α*,6*α*-triol (**43**) *	Antifouling and DNA top I inhibitory activity	Soft coral	[20]
Stigmasterol (**44**)	—	Soil	[21]
*β*-Sitosterol (**45**)	—	Soil	[21]
Ergosterol (**46**)	—	Soil	[21]
Trichosordarin A (**47**) *	Toxic to zooplankton	Sediment	[22]
Harzianumol A (**48**) *	—	Sponge	[23]
Harzianumol B (**49**) *	—	Sponge	[23]
Harzianumol C (**50**) *	—	Sponge	[23]
Harzianumol D (**51**) *	—	Sponge	[23]
Harzianumol E (**52**) *	—	Sponge	[23]
Harzianumol F (**53**) *	—	Sponge	[23]
Harzianumol G (**54**) *	—	Sponge	[23]
Harzianumol H (**55**) *	—	Sponge	[23]
Trichoharzin B (**56**) *	—	Soft coral	[20]
Methyl-trichoharzin (**57**) *	Antifouling	Soft coral	[20]
Trichoharzin (**58**) *	Antifouling	Soft coral	[20]
	—	Sponge	[29]
Eujavanicol A (**59**) *	—	Soft coral	[20]
Tandyukisin A (**60**) *	Cytotoxicity	Sponge	[11]
Tandyukisin B (**61**) *	Cytotoxicity	Sponge	[25]
Tandyukisin C (**62**) *	Cytotoxicity	Sponge	[25]
Tandyukisin D (**63**) *	Cytotoxicity	Sponge	[25]
Tandyukisin E (**64**) *	Cytotoxicity	Sponge	[24]
Tandyukisin F (**65**) *	Cytotoxicity	Sponge	[24]
Palmitic acid (**66**)	—	Soil	[21]
Harzianum A (**67**)	Antifungal	Soil	[26]
Harziphilone (**68**)	Cytotoxicity	Soil	[27]
Keto triol 3 (**69**)	Antifungal	Wheat roots	[28]
Keto diol 7 (**70**)	Antifungal	Wheat roots	[28]
Keto diol 6 (**71**)	Antifungal	Wheat roots	[28]
Keto diol 8 (**72**)	Antifungal	Wheat roots	[28]
Triacetate 9 (**73**)	Antifungal	Wheat roots	[28]
Triol 10 (**74**)	Antifungal	Wheat roots	[28]
Acetal diol 2 (**75**)	Antifungal	Wheat roots	[28]
Tribenzoate (**76**) *	—	Sponge	[29]
Triacetate (**77**) *	—	Sponge	[29]
T22azaphilone (**78**)	—	Commercial products	[30]
Trichoharzianol (**79**)	Antifungal	Soil	[31]
Trichodenone A (**80**) *	Cytotoxicity	Sponge	[32]
Trichodenone B (**81**) *	Cytotoxicity	Sponge	[32]
Trichodenone C (**82**) *	Cytotoxicity	Sponge	[32]
Homodimericin A (**83**)	—	Florida termite nest	[33,34]
Cryptenol (**84**)	—	Florida termite nest	[34]
Pachybasin (**85**)	—	Laboratory environment	[37]
Chrysophanol (**86**)	—	Laboratory environment	[37]
1,7-Dihydroxy-3-hydroxymethyl-9,10-anthraquinone (**87**)	Antifungal	Plant roots	[38]
1,5-Dihydroxy-3-hydroxymethyl-9,10- anthraquinone (**88**)	Antifungal	Plant roots	[38]
Emodin (**89**)	Antifungal	Plant roots	[38]
*ω*-Hydroxypachybasin (**90**)	Antifungal	Plant roots	[38]
Phomarin (**91**) *	—	Soft coral	[12]
(+)-2′*S*-Isorhodoptilometrin (**92**) *	Cytotoxicity	Soft coral	[12]
1,6-Dihydroxy-3-(hydroxymethyl)anthracene-9,10-dione (**93**) *	—	Soft coral	[12]
Harzianumnone A (**94**) *	—	Soft coral	[12]
Harzianumnone B (**95**) *	—	Soft coral	[12]
Trichokindin_Ia (**96**)	—	Soil	[40]
Trichokindin_Ib (**97**)	—	Soil	[40]
Trichokindin_IIa (**98**)	—	Soil	[40]
Trichokindin_IIb (**99**)	—	Soil	[40]
Trichokindin_IIIa (**100**)	—	Soil	[40]
Trichokindin_IIIb (**101**)	—	Soil	[40]
Trichokindin_IV (**102**)	—	Soil	[40]
Trichokindin_Va (**103**)	—	Soil	[40]
Trichokindin_Vb (**104**)	—	Soil	[40]
Trichokindin_VI (**105**)	—	Soil	[40]
Trichokindin_VII (**106**)	Induced catecholamine secretion	Soil	[40]
Harzianin_HB_I (**107**)	Membrane-modifying activity	Soil	[42]
Trichorzin_HA_I (**108**)	Antifungal	Soil	[43,44]
Trichorzin_HA_II (**109**)	Antifungal	Soil	[43,44]
Trichorzin_HA_III (**110**)	Antifungal	Soil	[43,44]
Trichorzin_HA_V (**111**)	Antifungal	Soil	[43,44]
Trichorzin_HA_VI (**112**)	Antifungal	Soil	[43,44]
Trichorzin_HA_VII (**113**)	Antifungal	Soil	[43,44]
Trichorzin_MA_I (**114**)	Antifungal	Soil	[43,44]
Trichorzin_MA_II (**115**)	Antifungal	Soil	[43,44]
Trichorzin_MA_III (**116**)	Antifungal	Soil	[43,44]
Trichorozin_I (**117**)	ion channel activity	Soil	[45]
Trichorozin_II (**118**)	ion channel activity	Soil	[45]
Trichorozin_III (**119**)	ion channel activity	Soil	[45]
Trichorozin_IV (**120**)	ion channel activity	Soil	[45]
Harzianin_HC_I (**121**)	Antibacterial	—	[46]
Harzianin_HC_III (**122**)	Antibacterial	—	[46]
Harzianin_HC_VI (**123**)	Antibacterial	—	[46]
Harzianin_HC_VIII (**124**)	Antibacterial	—	[46]
Harzianin_HC_IX (**125**)	Antibacterial	—	[46]
Harzianin_HC_X (**126**)	Antibacterial	—	[46]
Harzianin_HC_XI (**127**)	Antibacterial	—	[46]
Harzianin_HC_XII (**128**)	Antibacterial	—	[46]
Harzianin_HC_XIII (**129**)	Antibacterial	—	[46]
Harzianin_HC_XIV (**130**)	Antibacterial	—	[46]
Harzianin_HC_XV (**131**)	Antibacterial	—	[46]
Harzianin_PC_U_4 (**132**)	—	—	[47]
Trichorzin_PA_U_4 (**133**)	—	—	[47]
Trichorzin_PA_II (**134**)	—	—	[47]
Trichorzin_PA_IV (**135**)	—	—	[47]
Trichorzin_PA_V (**136**)	—	—	[47]
Trichorzin_PA_VI (**137**)	—	—	[47]
Trichorzin_PA_VII (**138**)	—	—	[47]
Trichorzin_PA_VIII (**139**)	—	—	[47]
Trichorzin_PA_IX (**140**)	—	—	[47]
Trichorzianine_TA_IIIc (**141**)	Anti-parasite	—	[48]
Trichorzianine_TB_IIa (**142**)	—	—	[49]
Trichorzianine_TB_IIIc (**143**)	—	—	[49]
Trichorzianine_TB_IVb (**144**)	—	—	[49]
Trichorzianine_TB_Vb (**145**)	—	—	[49]
Trichorzianine_TB_VIa (**146**)	—	—	[49]
Trichorzianine_TB_VIb (**147**)	—	—	[49]
Trichorzianine_TB_VII (**148**)	—	—	[49]
Trichodermamide G (**149**) *	—	Mangrove	[50]
Trichodermamide A (**150**) *	—	Mangrove	[50]
Aspergillazin A (**151**) *	—	Mangrove	[50]
Fleephilone (**152**)	Antivirus	Soil	[27]
Harzianic acid (**153**) *	Antibacterial	Water sample	[51]
Isoharzianic acid (**154**)	Plant growth promotion	Hardwood bark	[52]
Ethyl 2-bromo-4-chloroquinoline-3-carboxylate (**155**)	—	Soft coral	[20]
Harzianopyridone (**156**)	Antifungal	Soil	[21]
MR566A(**157**)	Melanin synthesis inhibition	Soil	[54,55]
MR566B (**158**)	Melanin synthesis inhibition	Soil	[54]
MR93A (**159**)	—	leaf	[53]
MR93B (**160**)	—	Soil	[54]
MR304A (**161**)	—	Soil	[54]
1-(1,4,5-Trihydroxy-3-isocyanocyclopenten-2-enyl)-ethanol (**162**)	—	Soil	[54]
2-Hydroxy-4-isocyano-*α*-methyl-6-oxabicyclo[3.1.0]-hex-3-ene-2-Methanol (**163**)	—	Soil	[54]
4-Hydroxy-8-isocyano-1-oxaspiro[4.4]cyclonon-8-en-2-one (**164**)	—	Soil	[54]
Methyl-3-(1,5-dihydroxy-3-isocyanocyclopent-3-enyl)prop-2-enoate (**165**)	—	Soil	[54]
3 -(3′-Isocyanocyclopent -2′-eny1idene)propionic acid (**166**)	Melanin synthesis inhibition	Soil	[54,55]
Xylogibloactone A (**167**) *	—	Soft coral	[20]
Xylogibloactone B (**168**) *	—	Soft coral	[20]
Nafuredin C (**169**) *	Antifungal	Mangrove	[50]
Nafuredin A (**170**) *	—	Mangrove	[50]
	Antifouling	Soft coral	[20]
Dichlorodiaportin (**171**) *	—	Soft coral	[20]
6-Pentyl-2*H*-pyran-2-one (**172**)	Antifungal	Soil	[21,58]
2(5*H*)-Furanone (**173**)	—	Soil	[21]
*δ*-Decanolactone (**174**)	—	Soil	[21]
Peniisocoumarin H (**175**) *	—	Mangrove	[50]
Harzialactone A (**176**) *	—	Sponge	[32]
Harzialactone B (**177**) *	—	Sponge	[32]
*R*-Mevalonolactone (**178**) *	—	Sponge	[32]
Harzianolide (**179**)	—	Commercial products	[30]
T39butenolide (**180**)	Antifungal	Commercial products	[30]

* Means marine source fungal strains.
